# Onset and duration of cycloplegic action of 1% cyclopentolate — 1% tropicamide combination

**DOI:** 10.4314/ahs.v17i3.36

**Published:** 2017-09

**Authors:** Samuel Kyei, Alfred Asiem Nketsiah, Kofi Asiedu, Agnes Awuah, Andrew Owusu-Ansah

**Affiliations:** 1 Department of Optometry, School of Allied Health Sciences, College of Health And Allied Science, University of Cape-Coast, Cape-Coast, PMB, Ghana; 2 Refraction and Low Vision Clinic , Eye Center, Komfo Anokye Teaching Hospital, P. O Box 1934, Kumasi, Ghana; 3 Department of Ophthalmology, The Second Xiangya Hospital of Central South University, Changsha, Hunan, China

**Keywords:** Cycloplegia, time course, accommodation, recovery time

## Abstract

**Purpose:**

To study the time course (onset, time and duration of maximal cycloplegia, and the full duration) of cycloplegic action of 1% Cyclopentolate — 1% Tropicamide.

**Methods:**

Seventy-seven students, aged 15–24 years were purposively sampled from the University of Cape Coast and Cape Coast Technical Institute. Subjective near addition (ADD) determination and pupil diameter measurement before and after a drop of the test agent (1% Cyclopentolate — 1% Tropicamide combination in the right eye) and the control (1% Cyclopentolate in the left eye) were performed. Measurements of subjective near ADD and pupil diameter were made after the initial reading at 5 minutes interval for the first hour and every 30 minutes for the next 7 hours for each participant. Time of onset, time of peak cycloplegia, duration of peak cycloplegia and duration of total cycloplegic effect was indirectly determined.

**Results:**

1% Cyclopentolate — 1% Tropicamide combination had rapid onset of cycloplegia (5–10 minutes), shorter time of maximal cycloplegia (55 versus 90 minutes), and recovery (7 versus ≥ 8hours) in the majority (79.2%) of subjects.

**Conclusion:**

Cyclopentolate -Tropicamide combination was comparable to Cyclopentolate in depth of cycloplegia produced, and clinically superior to 1% Cyclopentolate in rapidity of cycloplegic onset, time of maximal cycloplegia and recovery from cycloplegia.

## Introduction

Accurate determination of refractive status requires suppression of the accommodative response of the patient^1^. However, fogging with plus lenses is not always adequate, especially for the diagnosis of refractive conditions such as latent hyperopia^2^. Pharmacologic agents, known as cycloplegic agents, are used when such conditions are present or are suspected.

Cycloplegia refers to the pharmacological paralysis of the ciliary muscles, and it results primarily in inhibition of accommodation^2^,^3^. Cycloplegic agents prevent the action of acetylcholine at muscarinic receptor sites. Based on their mechanism of action, cycloplegics are referred to as anti-cholinergics, anti-muscarinics or parasympatholytic agents^4^,^5^. Muscarinic receptors are widely distributed throughout the human body, and immunoprecipitation studies have shown that all five muscarinic receptor subtypes are present in the human eye^6^, especially in the iris and ciliary body. Instillation of a cycloplegic agent results in inhibition of accommodation and also mydriasis (due to paralysis of the pupillae sphincter muscle). However, certain cycloplegics have been reported to have produced mydriasis but very little accommodative suppression^7^,^8^. Mydriasis also occurs without accompanying cycloplegia when a sympathomimetic agent is used. Thus, mydriasis is not always evidence of accompanying cycloplegia. Because of the different time courses of mydriasis and cycloplegia, pupil size is a poor indicator of the cycloplegic effect^4^,^9^.

Cycloplegic agents are indispensable in the diagnosis of latent hyperopia, pain relief from ciliary spasm, breaking or preventing irido-lenticular or irido-corneal adhesion, as well as penalization (or occlusion therapy) in eye care^3^,^10^–^12^. Cycloplegics also stabilize the blood-aqueous barrier and thus prevent further protein leakage (flares) into the anterior chamber^13^ in cases of uveitis. Though all the 5 muscarinic receptor sub-types are located in the eye, they are also widely distributed in other parts of the body. For this reason, anti-muscarinics are not only used in eye care^6^,^12^.

Generally, cycloplegics can cause local (ocular) and systemic side effects. Local effects include elevated intraocular pressure, blurred vision, photophobia, stinging sensation and injected bulbar conjunctiva^5^,^8^,^13^. Systemic side effects include dry mouth and skin, tachycardia, skin rash, psychotic reactions, behavioural disturbances, etc. Angle-closure glaucoma, after using an antimuscarinic agent, is a possible occurrence in susceptible persons but its incidence is however very rare^3^,^5^. Most adverse effects of antimuscarinics are associated with atropine use. They include hyperthermia, central nervous system (CNS) effects of hallucinations, restlessness, ataxia, and even death^3^,^8^,^12^. The ideal cycloplegic agent is one that has rapid onset, suppresses much accommodation, wears off faster, and is associated with no or minimal side effects. In response to finding an ideal cycloplegic agent, Cyclopentolate - Tropicamide combination has been reported by several researchers as having superior cycloplegic effect to the current agent of choice (Cyclopentolate) and comparable cycloplegic effect to Atropine, the gold standard^14^,^15^. The time course of action of Atropine, Cyclopentolate, and all other cycloplegics and mydriatic agents are well documented^3^,^8^,^16^.

Although Cyclopentolate — Tropicamide combination has been recommended as first line agent for cycloplegic examination, little or no information on the time course (onset, time to attain maximal cycloplegia, duration of maximal cycloplegia and total duration) of this combination is available for any racial population. This study focused on the time course of 1% Cyclopentolate — 1% Tropicamide combination among young African students in the Cape Coast metropolis of Ghana.

## Materials and methods

### Study design and subject selection

The study was conducted as an experimental study, to determine the time course of cycloplegic action of the test agent, i.e. 1% Cyclopentolate — 1% Tropicamide combination. Students were purposively recruited, and taken through an eye examination at the Department of Optometry clinic, University of Cape Coast. The examination involved;

- History taking to determine presence of any ocular or medical condition in which the use of a cycloplegic agent is contraindicated.
- Digital sphygmomanometry to determine subjects' normal blood pressure (Arm Digital Blood Pressure Monitor HZ-5915, Shanghai Huazhe Int. Co., Ltd, China).
- Distance and near visual acuity assessment (with Snellen and Grafton Optical near chart) for 6/6 and N.5 vision in each eye.
- Ocular health assessment including measuring normal intraocular pressure, assessing open or wide anterior chamber with Van Herrick's technique, and the absence of ocular disease using a slit lamp biomicroscope (YZ 5F1 Slit lamp with motorized table and applanation tonometer, Weihai Dingxin Optical Co., Ltd, China).

Subjects were excluded if they failed any of the criteria above.

One hundred and ten students were approached but after the examination, 77 students, comprising 27 females and 50 males aged 15 — 24 years, participated in the study to the end. Ten were excluded from the study after the initial examination as they did not meet the criteria indicated above. Another 4 did not turn up for the experimental study and 9 withdrew at various stages of the study.

### Data collection procedure

In order to minimize the effect of inter-subject variations on the results, the control (1 % Cyclopentolate) and test agents (1 % Cyclopentolate — 1% Tropicamide) were instilled in the left and right eye of the same subject respectively. Prospective participants were purposively sampled from University of Cape Coast and Cape Coast Technical Institute.

Prior to instillation of the drops, habitual pupil diameter of participants' eyes were measured (with a pupillometer), and least plus lens (subjective near ADD) through which N.5 optotypes on the Grafton Opticals near chart could be recognized and read were determined and recorded.

One drop of Drug A (1% Cyclopentolate — 1% Tropicamide combination) and Drug B (1% Cyclopentolate) were instilled in the lower fornix of each participant's anaesthetized right and left eye respectively, 2.5 minutes apart. This difference in time of instillation was to ensure that within the 5 minute period for each round of measurements, 2.5 minutes would be spent on each eye. After instillation, punctal occlusion was done in order to prevent or minimize the amount of drug that could get into systemic circulation.

Drug A was prepared by mixing an equal volume of 1% Cyclopentolate and 1% Tropicamide (Ashford Laboratories Ltd, China Macau). After instillation, the variables (pupil diameter and least near ADD) were measured every 5 minutes for 1 hour, and every 30 minutes for the next 7 hours. Data was collected from February 2016 to May 2016.

### Thin layer chromatography (TLC) of drug agents

In order to ascertain whether or not a new product was formed when equal volumes of 1% Cyclopentolate and 1% Tropicamide are mixed, thin layer chromatography was performed. The procedure is described below;

A straight line was drawn with a pencil along the breadth of a clean 10 cm x 3.6 cm preparative chromatographic plate (stationary phase) 1cm from the bottom edge of the plate. A drop of 1% Cyclopentolate, 1% Tropicamide and a mixture of the two compounds in 1:1 ratio were spotted on 1cm interval points along the straight line on the chromatographic plate, using clean capillary tubes. The spotted points were labelled; C for 1% Cyclopentolate, M for 1% Cyclopentolate — 1% Tropicamide mixture and T for 1% Tropicamide. Ethyl acetate (solvent system or the mobile phase) was poured into a clean dry jar so that it was 0.7cm deep from the bottom of the jar. The edge of the chromatographic plate, nearest to the spotted side was placed into the jar. After the paper was oriented to vertically lean against the wall of the jar, the jar was covered, and the setup was left undisturbed for a few minutes, during which the solvent rose to about ¾ of the length of the plate. The plate was then removed, dried and placed inside an iodine chamber for identification of compounds. The procedure was carried out at normal room temperature. The spotted points on the paper were then circled with a pencil, and measurements of distance moved by sample and solvent front were taken and their Rf values calculated^17^.

### Determination of pH

pH of the drug agents used was measured using a pH meter (Denver instrument GmbH, Germany). Each measurement was in triplicates and the mean ±SD (n=3) computed and recorded.

### Ethical considerations

The study was approved by the University of Cape Coast Institutional Review Board (Reference number: UCCIRB/CHAS/2015/065) and permission was sought from authorities of Cape Coast Technical Institute. Participants aged 18 years and older signed a written informed consent form which outlined the purpose of the study, procedures and its associated risks. Verbal consent and parental consent were obtained from participants below 18 years. Anonymity as well as confidentiality of participants was ensured. Participation in the study was voluntary, and participants could withdraw at any stage from the study. No incentives were provided to participants aside the free eye examination. The study was conducted in accordance with the tenets of the Helsinki declaration regarding the use of human subjects in research.

### Statistical analysis

Data was entered into and analyzed with Microsoft Excel 2013 and IBM SPSS Statistics 21 (The IBM Co., Chicago IL, USA.). Mean values of pupil diameter and least plus lens were calculated with Microsoft Excel 2013, and represented on graphs and tables. IBM SPSS Statistics 21 software was used for testing association; One sample T test was used for testing difference between the mean values for Drug A and Drug B, whereas Independent T test was used for testing difference between sub-groups' mean values (i.e. mean least plus lens values between males and females, and also between teenagers and young adults).

## Results

### Demographic characteristics

A total of 77 healthy students, 50 (65%) of which were males, and 27 (35%) were females participated in the study. Mean age of participants was 19.5 ± 2.48 years, with the youngest and oldest subject being 15 years and 24 years respectively. Two age groups were formed; 15–19 years group (teenage) and 20–24 years group (young adult), with the latter having the higher number of participants (43 subjects, 55.8%) in the study. The young adult group comprised 37 males and 6 females, with 12 males and 22 females in the teenage group.

### Mydriasis

**Fig. 1:**
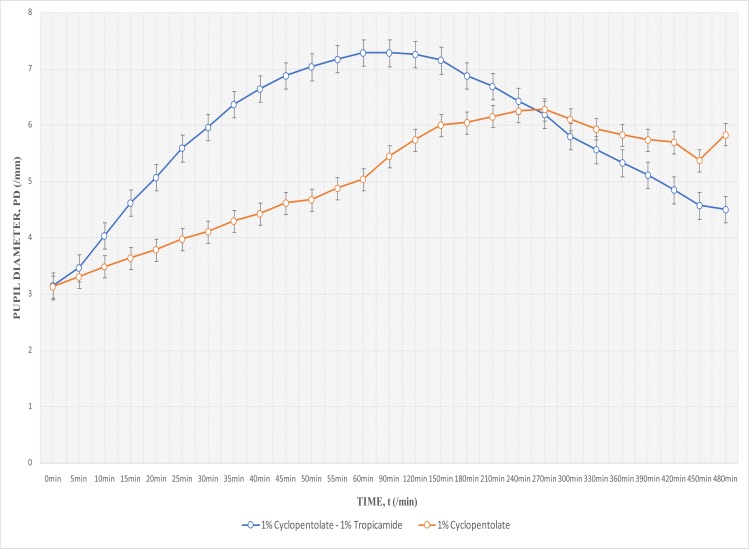
Pupil Diameter against Time for Drug A (1% Cyclopentolate- 1% Tropicamide) and Drug B (1% Cyclopentolate)

The mean habitual pupil diameter of the right and left eyes of all the subjects were 3.14 mm and 3.13 mm respectively (Fig. 1). A slight increase in pupil diameter was observed 5 minutes after instillation of 1% Cyclopentolate – 1% Tropicamide. However, the change of 0.32 mm that was measured is less than the least clinically-measurable change in pupil diameter of 0.5 mm. The first clinically-measurable change, i.e. at 3.65 mm pupil diameter, occurred 5–10 min after instillation, making this the clinically-significant onset of mydriasis of Drug A (1% Cyclopentolate — 1% Tropicamide). Clinically-significant onset of mydriasis of 1% Cyclopentolate(at 3.63 mm pupil diameter) occurred 15 min after instillation drops.

Significant differences were found in mydriatic action of Drug A and Drug B (p<0.001; One sample t test). Progression (increase) of mydriasis was observed to be rather slow with Drug B (1% Cyclopentolate). Peak mydriasis of 1% Cyclopentolate was 6.28 mm, and it occurred at 270 minutes. However, Drug A (1% Cyclopentolate - 1% Tropicamide) obtained 3.5 hours quicker and 1.1mm broader peak mydriasis (7.29 mm, at 60 min; Fig. 1).

Independent t test was run to find significant differences in mydriasis between males and females, and also between teenagers and young adults. There was no significant difference in mydriasis between males and females with either Drug A or B (p=0.45 for Drug A, p=0.54 for Drug B), and neither was there any significant difference between teenagers and the young adults (p=0.93 for Drug A, p=0.32 for Drug B).

### Cycloplegia

**Fig. 2:**
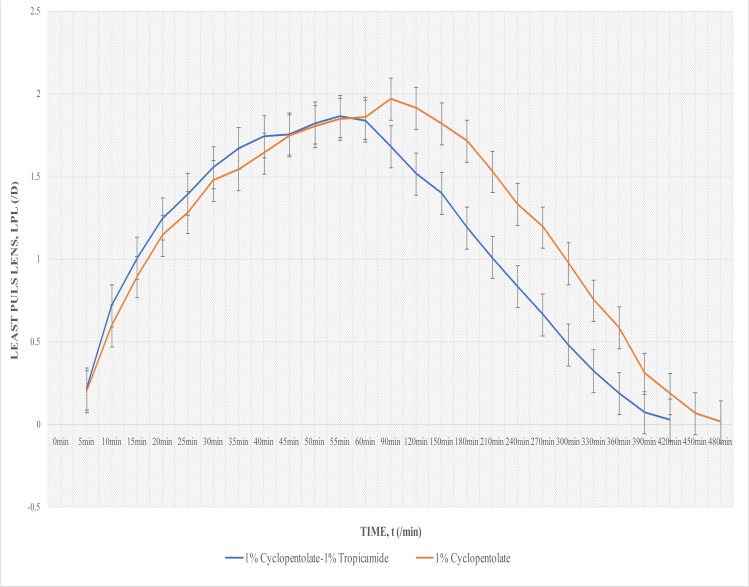
Least Plus Lens against Time for 1% Cyclopentolate- 1% Tropicamide combination and 1% Cyclopentolate

Prior to instillation, all participants had near visual acuity of N5 through plano lens. Clinically-measurable onset of cycloplegic action (requiring at least 0.25D plus lens), for both drugs, occurred 5 min after instillation (Fig. 2).

The summit of the least plus lens, (LPL) curve indicates peak cycloplegic activity. With 1% Cyclopentolate — 1% Tropicamide, LPL values increased and reached maximal cycloplegia at 55 min (1.85D). Taking into account that the refractive lens power equivalent for 1.85D lies between 1.75D and 2.00D, clinical equivalent of maximal cycloplegia is observed to have persisted for 55min (from 35–90 min, Fig. 2), and cycloplegic activity ended at 420 min (Fig. 2).

However, with 1% Cyclopentolate, maximal cycloplegia occurred at 90 min (1.96D ≈ 2.00D) and persisted for at least 30 minutes (from a time somewhere between 60 and 90 min to 120 min; Fig. 2). Cycloplegic activity of 1% Cyclopentolate ended at 480 min.

At their peaks, 1% Cyclopentolate achieved 0.11D more cycloplegia than 1% Cyclopentolate — 1% Tropicamide.

Majority of participants (61, 79.2%) recovered unaided reading ability (reading through plano lens) about 1 hour quicker in the eye instilled with 1% Cyclopentolate — 1% Tropicamide (Fig. 2). Generally, differences in the cycloplegic action of Drug A and Drug B were found to be significant (p<0.001; One sample t test).

**Fig. 3:**
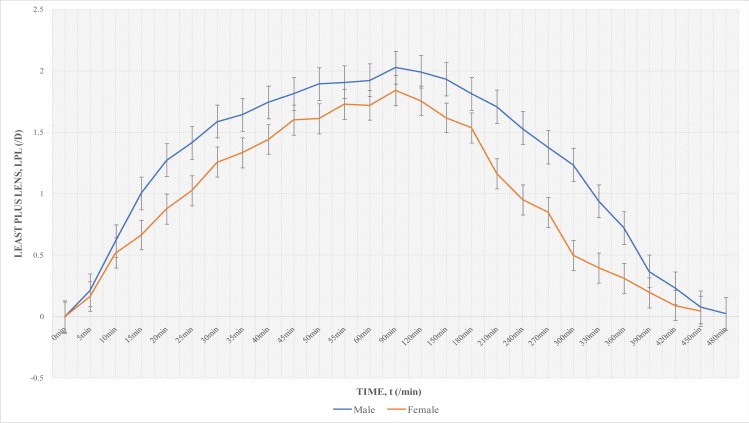
Gender Difference in Least Plus Lens with 1% Cyclopentolate

**Fig. 4:**
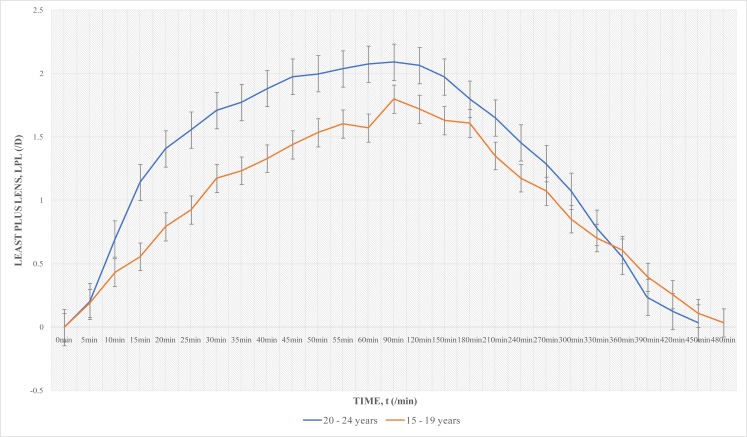
Age Difference in Least Plus Lens with 1% Cyclopentolate

Independent t test was run to find significant differences in cycloplegic action between males and females, and also between teenagers and young adults. With 1% Cyclopentolate – 1% Tropicamide, there were no significant difference in least plus lens between males and females (p=0.08), and between the teenagers and young adults (p=0.06). With 1% Cyclopentolate, there were significant differences in gender (p=0.02) and age (p=0.03) in least plus lens values. Females had lower values and about 30 mins shorter duration of total cycloplegic effect (Fig. 3). Subjects in the 15–19 years category also had lower values (of about 0.50D difference in clinical equivalent between them and the 20–24yrs category) from 15 min to 90min, and about 30 min longer duration than the young adult group (Fig. 4).

### Major variations

In 2 teenage females, mydriasis occurred without accompanying cycloplegia in the eye instilled with the combination drug and also in the eye instilled with 1% Cyclopentolate. In 1 young female adult, mydriasis occurred without accompanying cycloplegia in the eye instilled with 1% Cyclopentolate. A similar event occurred in a young male adult, howbeit in the eye instilled with the combination drug. In 1 young male adult, the eye instilled with 1% Cyclopentolate recovered unaided reading ability faster than the eye instilled with 1% Cyclopentolate — 1% Tropicamide. Finally, 10 subjects (made up of males and females belonging to the teenage and young adult group) recovered unaided reading ability in both eyes after 8 hrs.

### Thin layer Chromatography (TLC) result

**Fig. 5:**
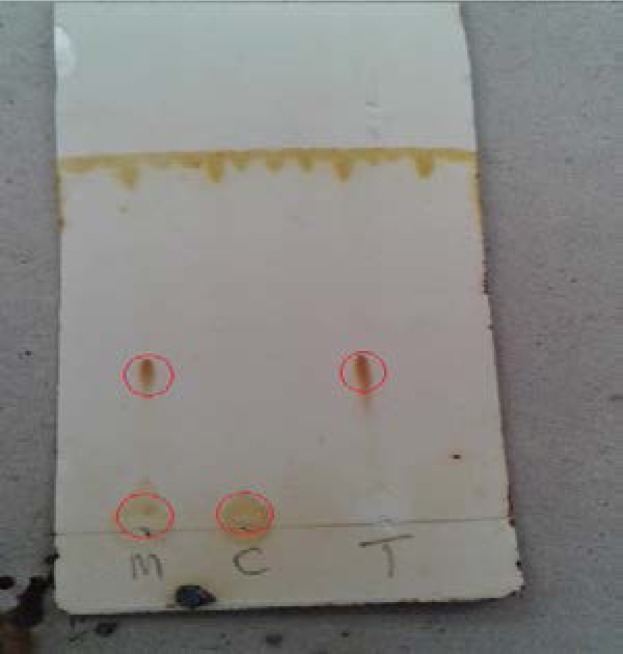
TLC plate showing spots

After the TLC procedure, 1% Tropicamide and 1% Cyclopentolate had one spot each with Rf values of 0.67 and 0.37 respectively (Figure 5). The mixture of 1% Cyclopentolate and 1% Tropicamide (1:1) had 2 spots and their Rf values were 0.37 and 0.67 respectively (Figure 5, marked as m). This compared to Rf values of the pure drug agents showed same Rf values to that of the combined agent, indicating that the combination had resulted in no new chemical agent.

**The pH recordings were as follows;**

- Cyclopentolate: 3.34 ± 0.03
- Tropicamide:4.56 ± 0.05
- Cyclopentolate — Tropicamide combination: 4.44 ± 0.04

### Side and adverse effects

Expected side effects, such as stinging on instillation and photophobia were reported by all participants. However, there was no report of any other adverse effect during or after the study.

## Discussion

Both the test and control drug agents had quick onset of mydriasis, but that of 1% Cyclopentolate — 1% Tropicamide was at least 5 minutes quicker. Tropicamide exerts an antagonistic effect on muscarinic receptors M4, M1 and M2, whereas Cyclopentolate antagonizes muscarinic receptor M1. Thus when combined, Tropicamide exerts an added effect of inhibiting M4 and M2, as Tropicamide and Cyclopentolate competitively inhibit M1 action^18^. This may be the reason why onset of mydriasis (5–10min) and duration of the combined drug (6–7 hrs) are similar to that of Tropicamide as their pHs were highly comparable and could have been better tolerated than the Cyclopentolate upon instillation suggesting a superior penetration of the ocular tissues^3^,^16^. Quicker onset of mydriasis in itself does not have much clinical implication, unlike rate of mydriatic progression (which affects time for maximal pupil dilation) and maximal pupil diameter. This is because dilated fundus examinations are performed on fully dilated pupils. 1% Cyclopentolate — 1% Tropicamide was observed to progress in mydriasis quicker, and attained peak mydrasis 3.5 hours before 1% Cyclopentolate did. With respect to dilated fundus exams, this means that all things being equal, patients administered with the combination drug would be examined 3.5 hours before those administered with 1% Cyclopentolate. This would ensure a significant and much appreciated decrease in waiting time for most patients and clinicians. In addition, there is a clinical advantage of being able to examine more of the vitreo-retinal space with 1% Cyclopentolate — 1% Tropicamide, during dilated intraocular assessment, since it was found to produce greater mydriasis (of 1mm more) than 1% Cyclopentolate.

Depth of cycloplegia was not the focus of this study. However, at their peaks, 1% Cyclopentolate achieved 0.11D more cycloplegia than 1% Cyclopentolate — 1% Tropicamide. The clinical equivalent for 0.11D (i.e. 0.00D) meant that 1% Cyclopentolate was not significantly superior to the combination drug in terms of the amount of cycloplegia that could be produced. The clinical implication is that, peak cycloplegia produced by 1% Cyclopentolate – 1%Tropicamide combination and 1% Cyclopentolate are comparable. Furthermore, considering clinical equivalent values, the greatest possible difference in peak cycloplegia was 0.25D. The capability, of one drug agent to produce a maximum of 0.25D peak cycloplegia in excess of that produced by another drug agent, would not be sufficient to recommend that drug over the other.

Peak mydriasis did not always coincide with peak cycloplegia. This has an important clinical implication; that maximal mydriasis should not be used as the only check for peak cycloplegia. Also in a few subjects, mydriasis occurred without clinically-significant cycloplegia. Possible reasons for this include tight squeezing of the lids immediately after instillation, (which resulted in loss of significant amount of drug) and/or increased reflex tears (as a result of stinging sensation) which resulted in loss of significant amount of drug and/or reduced concentration of drug. In both situations, the amount of drug that is absorbed would only be sufficient to cause mydriasis, but not cycloplegia. There are reports of certain concentrations of cycloplegics being effective for only mydriasis but not cycloplegia^7^,^8^. This may also be the explanation for the unusual finding of one young male subject recovering unaided reading ability faster with 1% Cyclopentolate. In any case, this study confirms a researcher's finding that pupil size is a poor indicator of cycloplegic effect^9^.

Ten participants (13%) comprising males and females across all the studied age groups regained unaided reading ability after 8 hrs. This was observed in eyes instilled with the combination drug, and also in eyes instilled with the single drug. Together with those who achieved mydriasis without accompanying cycloplegia, they form outliers of the study. An inherent anatomical or physiological phenomenon is probably the best explanation for such occurrences, since conditions for the study were equal for all subjects.

It is known that when melanin binds any drug, a lesser concentration of the unbound drug may be available to cause the pharmacologic effect in the ocular tissues. The pharmacological effect can be sustained over a relatively longer period of time by the slow release of the drug from melanin, if the binding is reversible. It is known that there are differences in melanin pigmentation between males and females, with females generally being lighter in skin pigmentation than males^19^. Thus if males are relatively much pigmented than females, it is understandable that a slower release of the drug (and consequently a longer duration of total cycloplegic effect) would be seen. It was therefore not surprising when females generally recovered unaided reading ability about 30 min faster with 1% Cyclopentolate. However, this cannot be considered the only explanation for this occurrence, because this was only seen with 1% Cyclopentolate, and not with the combination drug.

It has been reported that prior application of topical anesthesia can shorten the time to full cycloplegia,^20^ however, such influence would have masked the actual peak times in this comparative study. Several studies on cycloplegia did not administer topical anesthesia prior to instillation of cycloplegics^7^,^14^.

## Conclusion

Based on the findings of this study, it is our conclusion that the mixed agent provides a quicker method of cycloplegia, has an appreciable window of peak activity and wears off quicker, while offering as deep a cycloplegia as Cyclopentolate, and should be adopted for routine clinical use as it has the advantage of reducing waiting time. However further studies should be conducted into the adverse effect of its use.

## Figures and Tables

**Table 1:** Summary of Findings

FINDING	DRUG A	DRUG B
**ONSET OF MYDRIASIS**	5–10min	15min
**PEAK MYDRIASIS**	7.29mm	6.28mm
**TIME OF PEAK MYDRIASIS**	60min	270min
**ONSET OF CYCLOPLEGIA**	5min	5min
**TIME OF PEAK CYCLOPLEGIA**	55min	90min
**DURATION OF PEAK** **CYCLOPLEGIA**	55min (35–90min)	At least 30 minutes (90–120min)
**DURATION OF TOTAL** **CYCLOPLEGIC EFFECT**	7hr	≥ 8hr

Drug A: 1% Cyclopentolate — 1% Tropicamide Drug B: 1% Cyclopentolate
